# NSC-640358 acts as RXRα ligand to promote TNFα-mediated apoptosis of cancer cell

**DOI:** 10.1007/s13238-015-0178-9

**Published:** 2015-07-09

**Authors:** Fan Chen, Jiebo Chen, Jiacheng Lin, Anton V. Cheltsov, Lin Xu, Ya Chen, Zhiping Zeng, Liqun Chen, Mingfeng Huang, Mengjie Hu, Xiaohong Ye, Yuqi Zhou, Guanghui Wang, Ying Su, Long Zhang, Fangfang Zhou, Xiao-kun Zhang, Hu Zhou

**Affiliations:** School of Pharmaceutical Sciences, Xiamen University, Xiamen, 361102 China; School of Biological Science and Biotechnology, Minnan Normal University, Zhangzhou, 363000 China; Q-MOL LLC, San Diego, CA 92105 USA; Cancer Center, Sanford-Burnham Medical Research Institute, 10901 N. Torrey Pines Road, La Jolla, CA 92037 USA; Life Science Institute, Zhejiang University, Hangzhou, 310058 China; Institutes of Biology and Medical Sciences, Soochow University, Suzhou, 215123 China

**Keywords:** NSC-640358, ligand, RXRα, tRXRα, TNFα, apoptosis

## Abstract

Retinoid X receptor α (RXRα) and its N-terminally truncated version tRXRα play important roles in tumorigenesis, while some RXRα ligands possess potent anti-cancer activities by targeting and modulating the tumorigenic effects of RXRα and tRXRα. Here we describe NSC-640358 (N-6), a thiazolyl-pyrazole derived compound, acts as a selective RXRα ligand to promote TNFα-mediated apoptosis of cancer cell. N-6 binds to RXRα and inhibits the transactivation of RXRα homodimer and RXRα/TR3 heterodimer. Using mutational analysis and computational study, we determine that Arg316 in RXRα, essential for 9-*cis*-retinoic acid binding and activating RXRα transactivation, is not required for antagonist effects of N-6, whereas Trp305 and Phe313 are crucial for N-6 binding to RXRα by forming extra π–π stacking interactions with N-6, indicating a distinct RXRα binding mode of N-6. N-6 inhibits TR3-stimulated transactivation of Gal4-DBD-RXRα-LBD by binding to the ligand binding pocket of RXRα-LBD, suggesting a strategy to regulate TR3 activity indirectly by using small molecules to target its interacting partner RXRα. For its physiological activities, we show that N-6 strongly inhibits tumor necrosis factor α (TNFα)-induced AKT activation and stimulates TNFα-mediated apoptosis in cancer cells in an RXRα/tRXRα dependent manner. The inhibition of TNFα-induced tRXRα/p85α complex formation by N-6 implies that N-6 targets tRXRα to inhibit TNFα-induced AKT activation and to induce cancer cell apoptosis. Together, our data illustrate a new RXRα ligand with a unique RXRα binding mode and the abilities to regulate TR3 activity indirectly and to induce TNFα-mediated cancer cell apoptosis by targeting RXRα/tRXRα.

## INTRODUCTION

Retinoid X receptor α (RXRα), a unique member of the nuclear receptor superfamily, plays a pivotal role in diverse biological and physiological processes, including cell growth, differentiation, apoptosis, and homeostasis (Szanto et al., 2004; Evans and Mangelsdorf, 2014). RXRα has the typical structure of nuclear receptor, including an N-terminal region, a central DNA-binding domain (DBD), and a C-terminal ligand-binding domain (LBD) (de Lera et al., 2007). The unique property of RXRα among nuclear receptors lies in its ability to form heterodimers with many other nuclear receptors such as retinoic acid receptors (RARs) and peroxisome proliferator-activated receptors (PPARs) (Lefebvre et al., 2010; Evans and Mangelsdorf, 2014). Like other nuclear receptors, RXRα has important genomic functions mainly through its binding to responding DNA elements to regulate target gene transcription (Tang and Gudas, 2011; Evans and Mangelsdorf, 2014). Recently, accumulating evidence indicate that RXRα also resides in the cytoplasm to exhibit its non-genomic actions (Katagiri et al., 2000; Cao et al., 2004; Ghose et al., 2004; Zimmerman et al., 2006). For example, under certain stimuli, RXRα migrates from nucleus to cytoplasm with nerve growth factor IB (NGFIB, also known as Nur77 and TR3) to induce cell apoptosis by binding to Bcl-2 and converting Bcl-2 from an anti-apoptotic to a pro-apoptotic molecule (Li et al., 2000a; Cao et al., 2004; Lin et al., 2004).

Dysfunctions of RXRα are implicated in the initiation and development of a variety of diseases including cancer (Shulman and Mangelsdorf, 2005; Tang and Gudas, 2011). For examples, targeted disruption of *RXRα* gene leads to skin abnormalities and prostatic preneoplastic lesions (Li et al., 2000b; Huang et al., 2002), while abnormal phosphorylation of RXRα is associated with the progresses of colon cancer and liver cancer (Matsushima-Nishiwaki et al., 2001). The expression level of RXRα is often found reduced in cancer cells and tissues, implying the association of the less RXRα expression and carcinogenesis (Jiang et al., 1999; Lotan et al., 2000; Takiyama et al., 2004). A number of studies have shown that proteolytic cleavage of RXRα is one of the mechanisms for lower expression of RXRα in cancer cells (Nagaya et al., 1998; Nomura et al., 1999; Casas et al., 2003). Recently, we showed that calpain II cleaves RXRα to produce a truncated RXRα–tRXRα in cancer cells (Gao et al., 2013). Different from full-length RXRα, tRXRα is able to reside in the cytoplasm and interact with p85α, the subunit of phophoinositide 3-kinase (PI3K), which leads to the enhanced TNFα-induced AKT activation (Zhou et al., 2010; Wang et al., 2013). tRXRα-mediated activation of the TNFα/PI3K/AKT pathway significantly promotes cancer cell growth both *in vitro* and *in vivo* (Zhou et al., 2010; Wang et al., 2013), providing a potential approach to inhibit cancer cell growth by targeting tRXRα with small molecules to inhibit TNFα/PI3K/AKT survival pathway.

The functions of nuclear receptors are tightly and delicately regulated by their cognate ligands (Gronemeyer et al., 2004). A number of natural and synthetic compounds have been identified as RXRα selective ligands (Altucci et al., 2007; de Lera et al., 2007; Dawson and Xia, 2012). In general, the chemical structures of RXRα ligands, such as 9-*cis*-retinoic acid (9-*cis*-RA) and LG100754, contain an acidic moiety and a large hydrophobic moiety. The acidic moiety is able to form salt bridges with Arg316 residue in RXRα ligand binding pocket (LBP), which contributes immensely to ligand-receptor interaction (Altucci et al., 2007; Dawson and Xia, 2012). Comparison of RXRα apo (unliganded) and holo (liganded) crystal structures reveals a ligand-induced conformational change of RXRα, which significantly affects the interactions of RXRα with other proteins such as co-activators and co-repressors (de Lera et al., 2007). Although binding to the same LBP, different ligands induce distinct functions of RXRα, which is due to the distinct conformational changes of RXRα induced by different ligands (Altucci et al., 2007; de Lera et al., 2007; Perez et al., 2012). TR3 is an orphan nuclear receptor which can form heterodimer with RXRα (Moll et al., 2006). Crystal studies have shown that the LBD of TR3 lacks a typical hydrophobic pocket for accommodating ligands (Wang et al., 2003). However, TR3 is able to recruit co-activator for transactivation independent of ligand binding (Wansa et al., 2002), reflecting its active unliganded conformation resembling other agonist-activated nuclear receptors.

In this study, we show that NSC-640358 (N-6) is a selective RXRα antagonist with a unique RXRα binding mode. Through binding to RXRα, N-6 inhibits RXRα transactivation and RXRα-mediated transcriptional activity of TR3. Moreover, N-6 inhibits tumor necrosis factor α (TNFα)-induced AKT activation and enhances TNFα-mediated cancer cell apoptosis in an RXRα/tRXRα dependent manner.

## Results

### N-6 binds to RXRα

**Figure 1:**
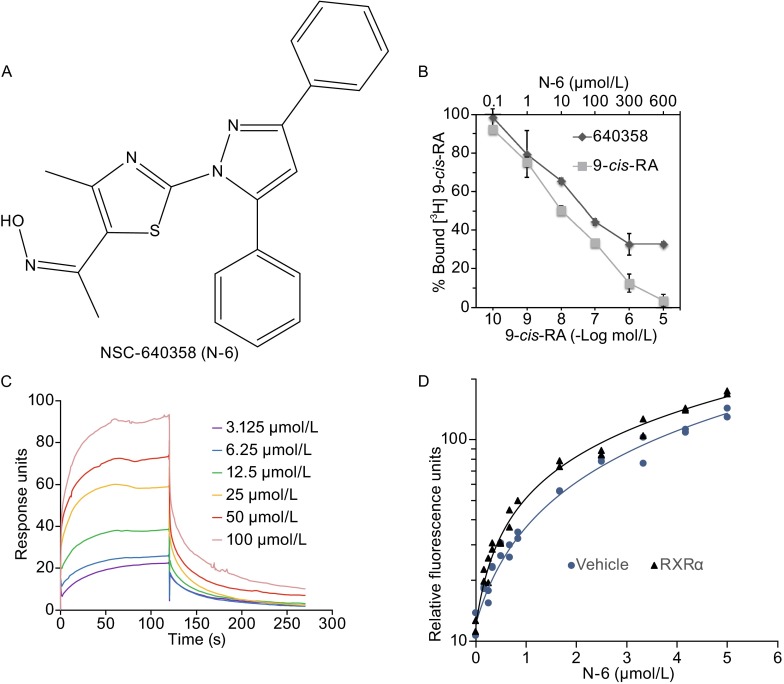
**N-6 binds to RXRα**. (A) Structure of NSC-640358 (N-6). (B) RXRα-LBD protein was incubated with [^3^H]9-*cis*-RA in the presence or absence of unlabeled 9-*cis*-RA or N-6. Bound [^3^H]9-*ci*s-RA was quantitated by liquid scintillation counting. (C) Gradient concentrations of N-6 were injected through flow cells immobilized with RXRα-LBD. The kinetic profiles are shown and the dissociation constant (*K*_d_) of the N-6/RXRα-LBD complex was calculated to be 15.755 × 10^−6^ mol/L. (D) RXRα-LBD protein enhanced fluorescent intensity of N-6 (ex 278 nm, em 338 nm, cutoff 325 nm, delay 50 μs, integration 450 μs). One of three similar experiments is shown

**Figure 2:**
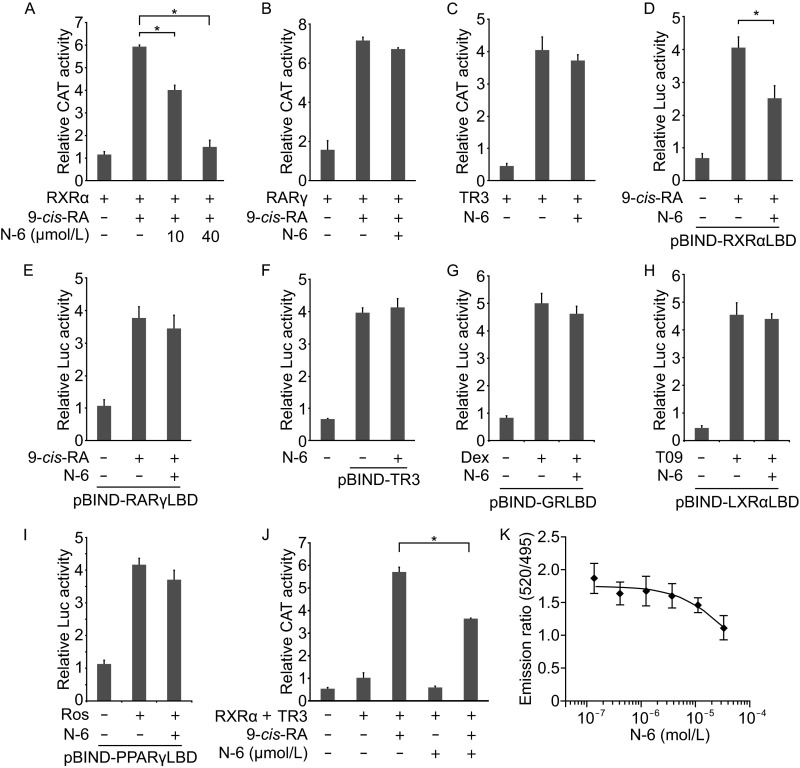
**N-6 is a selective antagonist of RXRα**. (A–C) CV-1 cells were cotransfected with (TREpal)_2_-*tk*-CAT and pCMV-Myc-RXRα (A), (TREpal)_2_-*tk*-CAT and pCMV-Myc-RARγ (B), or NurRE-*tk*-CAT and pCMV-Myc-Nur77 (C). Cells were treated with N-6 (10 μmol/L) and 9-*cis*-RA (10^−7^ mol/L) for 18 h. CAT activities were measured and normalized to β-galactosidase activities (*P < 0.05). (D–I) MCF-7 cells were cotransfected with pG5-Gaussia-Dura and pBIND-RXRαLBD (D), pBIND-RARγLBD (E), pBIND-TR3 (F), pBIND-GRLBD (G), pBIND-LXRαLBD (H), or pBIND-PPARγLBD (I). Cells were treated with N-6 (10 μmol/L) in the presence or absence of 0.1 μmol/L 9-*cis*-RA (D and E), 1 μmol/L Dexamethasone (Dex) (G), 1 μmol/L T0901317 (T09) (H), or 1 μmol/L Rosiglitazone (Ros) (I) for 18 h. Reporter activities were measured and normalized (*P < 0.05). (J) CV-1 cells transfected with βRARE-*tk*-CAT, RXRα and TR3 expression vectors were treated with N-6 (10 μmol/L) in the presence or absence of 9-*cis*-RA (10^−7^ mol/L) for 18 h. CAT activities were measured and normalized to β-galactosidase activities (*P < 0.05). (K) GST-RXRα-LBD was incubated with different concentrations of N-6 in the presence of 9-*cis*-RA (10^−8^ mol/L) for 2 h. FRET signals were measured and normalized. The IC_50_ of N-6 was calculated to be 3.29 × 10^−5^ mol/L. One of three similar experiments is shown. Data shown are mean ± SD

**Figure 3:**
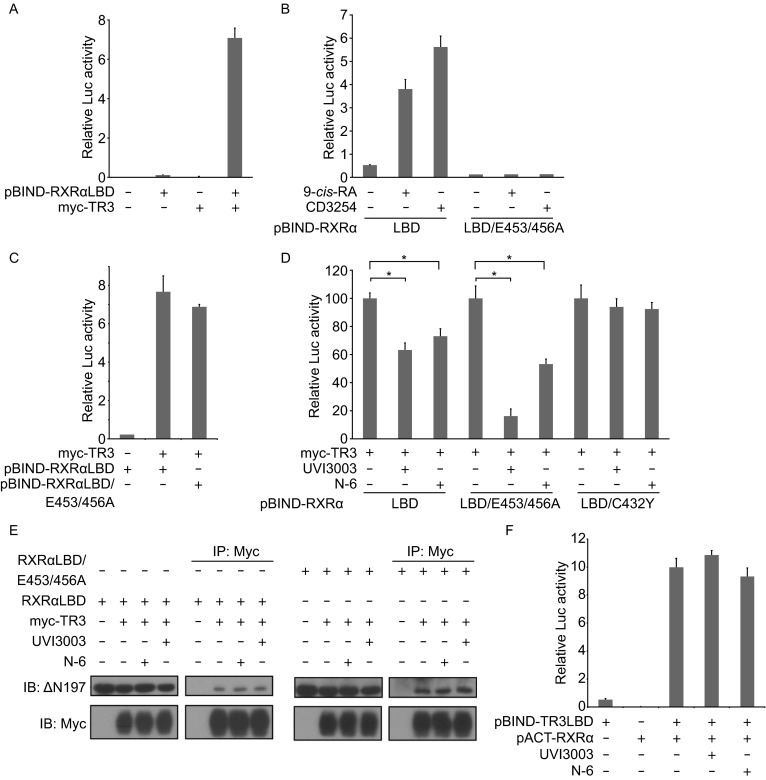
**N-6 inhibits TR3 transcriptional activity by binding to RXRα**. (A–D, and F) MCF-7 cells cotransfected with pG5-Gaussia-Dura reporter vector and the indicated expression vectors were treated with or without N-6 (10 μmol/L), UVI3003 (1 μmol/L), and 9-*cis*-RA (10^−7^ mol/L) for 18 h. Reporter activities were measured and normalized. Data shown are mean ± SD (*P < 0.05). (E) HEK293T cells cotransfected with pCMV-Myc-TR3 and pBIND-RXRα-LBD expression vectors were treated with UVI3003 (1 μmol/L) or N-6 (10 μmol/L) for 18 h. Cell lysate were analyzed for heterodimerization of Nur77 and Gal4-DBD-RXRα-LBD by co-immunoprecipitation with anti-myc antibody. One of three similar experiments is shown

**Figure 4:**
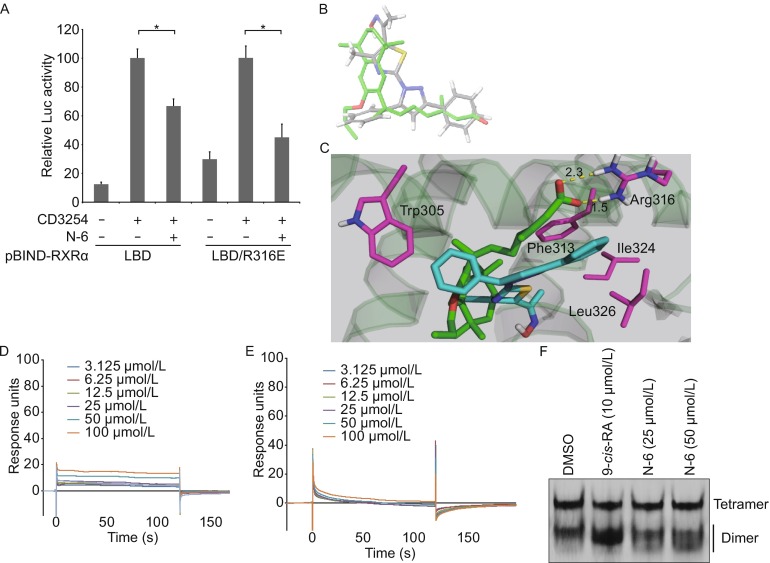
**Arg316 is not required for N-6 binding to RXRα**. (A) MCF-7 cells cotransfected with pG5-Gaussia-Dura reporter vector and pBIND-RXRα-LBD or pBIND-RXRα-LBD/R316E expression vectors were treated with or without N-6 (10 μmol/L) in the presence or absence of CD3254 (10^−7^ mol/L) for 18 h. Reporter activities were measured and normalized. Data shown are mean ± SD (*P < 0.05). (B) Comparison of the docked conformation of N-6 (gray) with the crystal structure of LG100754 (green). (C) N-6 was docked into the LBP of the co-crystal structure of LG100754 and RXRα-LBD (PDB 3A9E). Salt bridges are shown as dotted yellow lines, and residues interacting with N-6 are shown in magenta. (D–E) Gradient concentrations of N-6 were injected through flow cells immobilized with RXRα-LBD/Trp305Ala (D) and RXRα-LBD/Phe313Ala (E), respectively. The kinetic profiles are shown and the dissociation constants (*K*_d_) of the N-6/RXRα-LBD complex were calculated to be 1.0 × 10^−3^ mol/L (D) and more than 1.0 × 10^−2^ mol/L (E). (F) RXRα-LBD proteins (2 mg/mL) was incubated with DMSO, 10 μmol/L 9-*cis*-RA, 25 μmol/L N-6 or 50 μmol/L N-6 for 3 h, and proteins were separated by 8% nondenaturing PAGE followed by Commassie Blue staining

**Figure 5:**
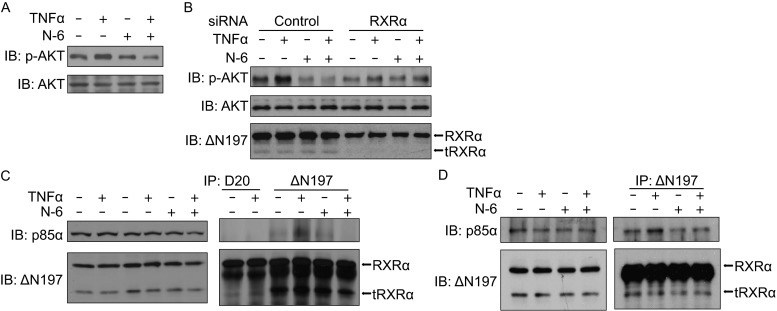
**N-6 inhibits TNFα-induced AKT activation in a tRXRα-dependent manner**. (A) HCT116 cells were pretreated with N-6 (10 μmol/L) for 2 h in serum free medium before being exposed to TNFα (10 ng/mL) for an additional 30 min. Lysates prepared were analyzed by Western blotting for AKT activation. (B) A549 cells transfected with RXRα siRNA or control siRNA for 48 h were treated with N-6 (10 μmol/L) for 2 h in serum free medium before being exposed to TNFα (10 ng/mL) for an additional 30 min. Lysates prepared were analyzed by Western blotting for AKT activation. (C and D) H292 cells (C) or A549 cells (D) pretreated with N-6 (10 μmol/L) for 2 h in serum free medium before being exposed to TNFα (10 ng/mL) for an additional 30 min were analyzed for p85α/tRXRα interaction by co-immunoprecipitation assay using anti-RXRα antibodies of D20 or ΔN197. Immunoprecipitates were analyzed by Western blotting for the presence of p85α and tRXRα. One of three similar experiments is shown

**Figure 6:**
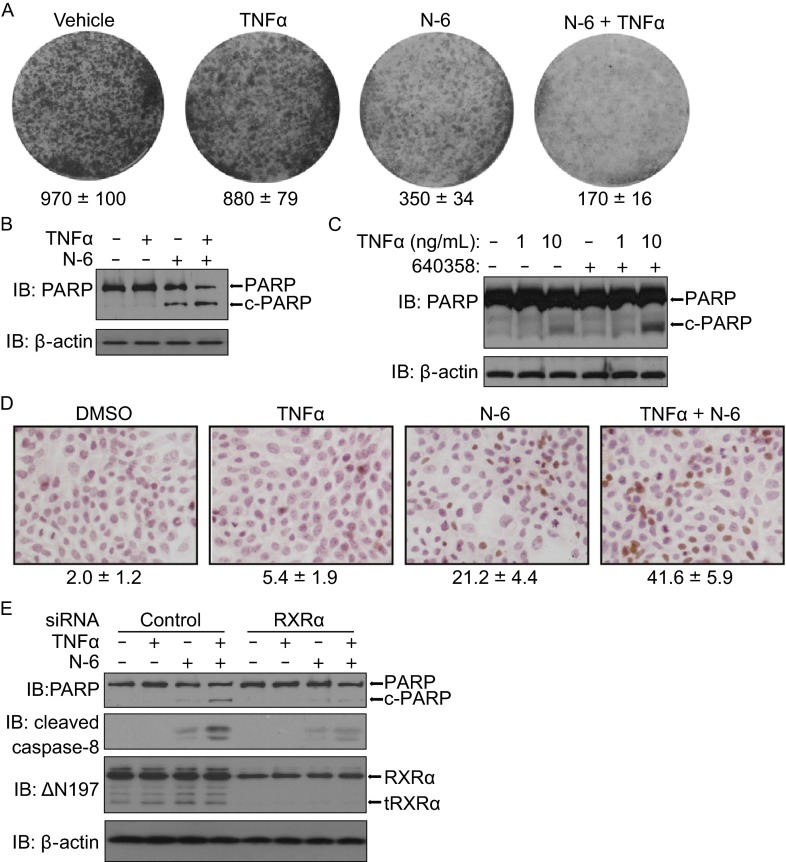
**Combination of TNFα and N-6 induces cancer cell apoptosis in an RXRα/tRXRα-dependent manner**. (A) HeLa cells grown in 6-well plates were treated with or without N-6 (5 μmol/L) and TNFα (10 ng/mL) for 3 days. Colonies were stained with 0.1% crystal violet and counted. (B and C) HCT116 and MCF-7 cells were treated with or without N-6 (10 μmol/L) and TNFα (10 ng/ mL) for 15 h in serum free medium. Cell lysates prepared were analyzed by Western blotting for PARP cleavage. (D) MCF-7 cells grown in 24-well plates were treated with or without N-6 (10 μmol/L) and/or TNFα (10 μg/mL) for 3 days. Apoptotic cells were detected by TUNEL staining and counted. (E) HCT116 cells transfected with RXRα siRNA or control siRNA for 48 h were treated with or without N-6 (10 μmol/L) and TNFα (10 ng/mL) for 15 h. Cell lysates prepared were analyzed by Western blotting for PARP and caspase-8 cleavage. One of three similar experiments is shown

We used Q-MOL molecular modeling package to perform a virtual screening of a compound library for RXRα ligands, and found that NSC-640358 ((NZ)-N-[1-[2-(3,5-diphenylpyrazol-1-yl)-4-methyl-1,3-thiazol-5-yl]ethylidene]droxylamine, N-6), a thiazolyl-pyrazole derived compound, was a potential RXRα ligand (Fig. 1A). The interaction of N-6 and RXRα was first examined by ligand competition binding assay. As a positive control, unlabeled 9-*cis*-RA displaced [^3^H]-labeled 9-*cis*-RA for binding to RXRα-LBD protein in a dose-dependent manner (Fig. 1B). Similarly, N-6 dose-dependently competed with [^3^H]-labeled 9-*cis*-RA for binding to RXRα-LBD protein with an IC_50_ at 11.3 μmol/L (Fig. 1B), suggesting the direct binding of N-6 to RXRα. Then we performed surface plasmon resonance (SPR) technology-based experiment to confirm their physical binding. As shown in Fig. 1C, N-6 dose-dependently binds to RXRα-LBD with a *K*_d_ value of 15.7 μmol/L. Furthermore, our fluorescence ligand binding assay showed that RXRα-LBD protein potently enhanced the fluorescence intensity of N-6 (Fig. 1D). Together, these results demonstrate that N-6 is able to bind to RXRα directly.

### N-6 selectively inhibits RXRα transactivation

To examine whether N-6 regulated RXRα transactivation, a reporter gene containing RXRα homodimer-responsive elements, (TREpal)_2_-*tk*-CAT (Dawson et al., 2001), was transfected with a RXRα expression vector into CV-1 cells that lack detectable levels of endogenous RXRα (Zhang et al., 1992a, b). Treatment of cells with 9-*cis*-RA potently induced the transactivation of RXRα homodimer, which was dose-dependently inhibited by N-6 (Fig. 2A). In contrast, N-6 failed to inhibit 9-*cis*-RA-induced transactivation of RARγ homodimer or the ligand-independent transactivation of Nur77 (Fig. 2B and 2C) (Kolluri et al., 2003). Thus, N-6 selectively inhibits RXRα transactivation, which was further confirmed by our mammalian one-hybrid technology-based assay. N-6 strongly inhibited 9-*cis*-RA-induced Gal4-DBD-RXRα-LBD transactivation but not the other chimeric nuclear receptors, such as RARγ, TR3, GR, LXRα and PPARγ (Fig. 2D–I). We next examined the effects of N-6 on the transcriptional activity of RXRα/TR3 heterodimer. To this end, CV-1 cells were transfected with RXRα and TR3 expression vectors together with βRARE-*tk*-CAT reporter. 9-*cis*-RA-induced transactivation of RXRα/TR3 heterodimer was strongly inhibited by N-6 (Fig. 2J). It is known that the binding of 9-*cis*-RA induces RXRα conformational change, leading to co-activator recruitment. We then examined whether N-6 inhibited 9-*cis*-RA-induced co-activator recruitment. As shown in Fig. 2K, N-6 dose-dependently inhibited 9-*cis*-RA-induced interaction of co-activator and RXRα-LBD with an IC_50_ at 32.9 μmol/L. Taken together, these findings indicate that N-6 is a selective antagonist of RXRα.

### N-6 regulates TR3 activity by binding to RXRα

In the absence of RXRα agonists, the fusion protein Gal4-DBD-RXRα-LBD only exhibited basal transactivation. However, transfection of TR3 remarkably enhanced its transactivation (Fig. 3A). The salt bridges formed by Glu453/456 in helix 12 and Arg302 in helix 4 are essential for maintaining co-activator binding site in RXRα (Egea et al., 2000; Egea et al., 2002). The fusion protein Gal4-DBD-RXRα-LBD/E453/456A, with Ala substituted with Glu, completely lost transactivation ability in response to 9-*cis*-RA and CD3254 (Fig. 3B). However, TR3 was still able to stimulate the reporter gene activity together with Gal4-DBD-RXRα-LBD/E453/456A (Fig. 3C). Sequence analysis indicated there were no cognate TR3 binding sites in the G5 reporter vector. Thus, the ability of TR3 to induce the transactivation was due to the interaction of Gal4-DBD-RXRα-LBD with TR3 and TR3 ligand-independent transactivation. No matter what above fusion proteins were used, TR3-induced transactivation was potently inhibited by N-6 (Fig. 3D). However, in the case of Gal4-DBD-RXRα-LBD/C432Y with disrupted RXRα-LBP due to the substitution of Cys with Tyr, N-6 completely lost its activity (Fig. 3D), suggesting the necessity of the binding of N-6 to RXRα-LBD. Similar results were observed when RXRα antagonist UVI3003 was examined (Fig. 3D). Thus, N-6 and UVI3003 bind to RXRα to indirectly inhibit TR3 transactivation. One possible mechanism by which N-6 inhibited TR3 transactivation was the disruption of the interactions of TR3 with the fusion proteins, which was analyzed by our co-immunoprecipitation experiments. As shown in Fig. 3E, Myc-TR3 interacted with the fusion proteins, as evidenced by their co-precipitation. Neither N-6 nor UVI3003 affected their interactions (Fig. 3E). GAL4-DBD-TR3-LBD had low, if there was, transactivation activity, which was due to the loss of the N-terminal TR3 (Wansa et al., 2002). When both GAL4-DBD-TR3-LBD and p16-ACT-RXRα fusion proteins existed, a dramatic induction of the reporter gene activity was observed, which reflected a typical mammalian two-hybrid assay to show the interaction of RXRα and TR3-LBD. In this context, neither N-6 nor UVI3003 was able to inhibit the induced transactivation (Fig. 3F), which also demonstrated the inability of N-6 and UVI3003 for blocking the interaction of TR3 and RXRα. Therefore, it is not through the disruption of the interaction of TR3 and RXRα for N-6 to inhibit TR3 transactivation.

### N-6 binds to RXRα in a distinct mode

Unlike many natural and synthetic RXRα ligands, N-6 lacks a carboxylate moiety known to form salt bridges with Arg316 in RXRα-LBP. To examine the requirement of Arg316 for N-6, it was replaced with Glutamic acid, and the resulting mutant Gal4-DBD-RXRαLBD/R316E was evaluated for its transactivation regulated by N-6. As shown in Fig. 4A, CD3254 was able to stimulate the transactivation of the mutant. Similar to RXRα-LBD, CD3254-induced transactivation of RXRα-LBD/R316E was potently inhibited by N-6 (Fig. 4A), implying that Arg316 was not required for N-6 binding to RXRα. We then used computer-aided and docking-based techniques to analyze the binding mode of N-6. In light of the antagonist activity of N-6 and a large degree of structural overlapping between N-6 and LG100754 (Fig. 4B), an antagonist of RARα/RXRα heterodimer (Sato et al., 2010), it was reasonable that the model of RXRα under its antagonist conformation should be used for the docking of N-6. In fact, our docking study showed that N-6 was well accommodated into the LBP of RXRα with an antagonist conformation (Fig. 4C). Unlike LG100754, N-6 did not form ionic bonds (2.3 Å and 1.5 Å for LG100754) with Arg316. However, N-6 possesses two aromatic rings that could establish extra π-π stacking interactions with Phe313 and Trp305 (Fig. 4C). The essential role of Phe313 and Trp305 in N-6 binding was confirmed by our SPR assays, showing that N-6 had much lower binding affinity to two RXRα-LBD point mutants with Ala substitution of Phe313 or Trp305 (Fig. 4D and 4E). Furthermore, our docking study showed that two hydrophobic residues Ile324 and Leu326 could produce additional hydrophobic interactions with N-6 (Fig. 4C). Ligand binding often leads to the conformational change of RXRα protein, which was investigated for N-6 by our native gel electrophoresis assay. Consistent with previous reports, 9-*cis*-RA strongly induced the homodimeric formation of RXRα-LBD protein. However, N-6 dose-dependently induced the conformational changes of RXRα-LBD dimer, indicated by the pattern changes of the dimer bands (Fig. 4F). Taken together, our data indicate that N-6 has a distinct binding model comparing with classic RXRα ligands.

### N-6 inhibits TNFα-induced AKT activation in an RXRα/tRXRα dependent manner

It has been reported that RXRα antagonist K-80003 inhibits TNFα-induced AKT activation in a tRXRα-dependent pathway (Zhou et al., 2010). Here we examined whether N-6 had a similar function. Figure 5A showed that N-6 significantly inhibited TNFα-induced AKT activation in HCT116 cells, revealed by its suppression of the expression of phosphor-AKT but not total AKT (Fig. 5A). Similar results were obtained in A549 cells (Fig. 5B). We then determined the requirement of RXRα/tRXRα for the inhibition of AKT activation by N-6. Transfection of RXRα siRNA in A549 cells, which reduced both the full-length RXRα and tRXRα expression, abolished the inhibitory effect of N-6 on TNFα-induced AKT activation (Fig. 5B). Thus, the expression of RXRα/tRXRα is essential for AKT inhibition by N-6. We have reported that K-80003 inhibited AKT activation by blocking the TNFα-induced interaction of tRXRα and p85α (Zhou et al., 2010). We then investigated whether N-6 had the similar action. Two anti-RXRα antibodies, ∆N197 and D20, were used in the co-immunoprecipitation assay, of which ∆N197 but not D20 could recognize both RXRα and tRXRα (Zhou et al., 2010). Consistent with our previous reports (Zhou et al., 2010), TNFα strongly induced the interaction of tRXRα but not RXRα with p85α showed by our co-immunoprecipitation experiment (Fig. 5C). However, when cells were treated with N-6 together with TNFα, the interaction of tRXRα and p85α promoted by TNFα was completely blocked (Fig. 5C and 5D). Thus, the inhibitory effect of N-6 on TNFα-induced AKT activation might rely on its disruption of the interaction of tRXRα with p85α.

### N-6 induces RXRα/tRXRα-dependent apoptosis of cancer cell

The ability of N-6 to inhibit AKT activation prompted us to examine the effect of N-6 on the survival of cancer cells by colonogenic survival assay. Treatment of cells with N-6 significantly inhibited colony formation of HeLa cells, which was dramatically enhanced by TNFα (Fig. 6A). The inhibition of TNFα-induced AKT activation often leads to the activation of TNFα-mediated apoptosis (Zhou et al., 2010; Wang et al., 2013). Indeed, the combination of TNFα and N-6 synergistically induced PARP cleavage in HCT116 cells (Fig. 6B). The synergistic induction of PARP cleavage was TNFα-concentration dependent in MCF-7 cells (Fig. 6C). Furthermore, our TUNEL staining assay confirmed the synergistic pro-apoptosis of the combination (Fig. 6D). We then determined the role of RXRα/tRXRα in apoptosis induction of the combination using knocking down approach. Compared to control siRNA, RXRα siRNA impaired the ability of the combination of N-6 and TNFα for the induction of PARP cleavage (Fig. 6E). Accompanying with PARP cleavage, the combined treatment also induced caspase-8 activation, which was also inhibited by the suppression of RXRα/tRXRα expression (Fig. 6E). Thus, RXRα/tRXRα is involved in the apoptotic induction of the combination of N-6 and TNFα.

## Discussion

RXRα is a promising drug target for cancer therapy, as it has an inner LBP to accommodate “drug-like” small molecule modulators (Altucci et al., 2007; de Lera et al., 2007). In addition, dysfunctions or abnormal expressions of RXRα are implicated in tumor development (Tang and Gudas, 2011; Thomas et al., 2012). Recently, we reported that RXRα antagonist K-80003 exhibits striking anti-cancer effects by inhibiting the cytoplasmic activation of PI3K/AKT by tRXRα (Zhou et al., 2010). Here, we present a new RXRα ligand with a distinct structure and an RXRα/tRXRα-dependent induction of cancer cell apoptosis with TNFα.

The structure of N-6 is very different from known RXRα ligands such as 9-*cis*-RA and LG100754. However, it bound to RXRα with a unique mode and acted as an RXRα antagonist (Figs. 1, 2 and 4). The holo-crystal structure of 9-*cis*-RA and RXRα-LBD indicates that the salt bridge formed via Arg316 at the end of the L-shaped LBP and the carboxylate group of 9-*cis*-RA is crucial for 9-*cis*-RA binding to RXRα (Egea et al., 2000; Egea et al., 2002; Sato et al., 2010). N-6 lacks such a carboxylate moiety, and therefore it should not require Arg316 for binding, which was verified by our mammalian one-hybrid assay showing that the mutation of Arg316 did not affect the inhibition of RXRα transactivation by N-6 (Fig. 4A). Recently, several compounds without carboxylate moiety such as CF31, Danthron, Magnolol, and Bigelovin were reported to bind to RXRα (Zhang et al., 2011a; Zhang et al., 2011b; Zhang et al., 2011c; Wang et al., 2013). Therefore, salt bridges are not necessary for some ligands binding to RXRα. Instead, in the case of N-6, the π-π stacking interactions and hydrophobic interactions contribute to the binding of N-6 to RXRα (Fig. 4C–E). N-6, CF31, and Danthron are RXRα antagonists while Magnolol and Bigelovin are RXRα agonists. Both as antagonist of RXRα, Danthron and N-6 might use different antagonistic mechanisms. In contrast with stabilization of RXRα tetramer by Danthron (Zhang et al., 2011c), N-6 might induce the conformational changes of RXRα dimer to inhibit its homodimer transactivation (Figs. 2A and 4F). The different mechanisms should be due to the distinct spatial structures and binding modes of Danthron and N-6 that lead to different conformational changes of RXRα. It will be of great interests to study how distinct RXRα conformational changes induced by these compounds without acidic group define their regulation properties and mechanisms of RXRα activities.

Crystal structure of TR3 indicates that it lacks a normal LBP (Wang et al., 2003). Although many compounds have been identified to bind to TR3 (Lee et al., 2011), the controversy of the cognate ligands of TR3 still exists. Thus, it might be difficult to design and apply strategy to regulate TR3 physiopathological functions by small molecules directly targeting TR3. An alternative approach is to use small molecules to regulate TR3 functions indirectly by targeting TR3 interacting proteins. Our study provides an example to show the feasibility of this approach. In the absence of binding to its cognate DNA elements, the transactivation of TR3 associated with RXRα was able to be inhibited by N-6 through binding to RXRα (Fig. 3D). The conformational change of RXRα induced by N-6 binding may enhance RXRα recruiting co-repressors that would prevent co-activator recruitment of TR3 due to steric hindrance, or may affect the activating conformation of TR3, especially its AB domain, in the RXRα/TR3 heterodimer. The activities of TR3 modulated by N-6 may not be limited to its transcriptional activities. Besides regulating gene transcription, TR3 also interacts with many proteins to exert its non-genomic functions in the absence of DNA binding (Lin et al., 2004; Moll et al., 2006). Therefore, our study also provides a potential strategy to use small molecules to target RXRα to regulate TR3 non-genomic functions.

The physiological functions of ligands often depend on their abilities of inducing conformational changes of their cognate nuclear receptors followed by the changes of interactions of nuclear receptors with other proteins. The conformational change of tRXRα induced by N-6 disrupted the interaction of tRXRα and p85α, leading to the inhibition of TNFα-induced AKT activation (Fig. 5). However, in the case of the interaction of RXRα and TR3, N-6 did not disrupt the interaction but definitely changed the interaction manner, leading to the inhibition of TR3 transactivation (Fig. 3). Therefore, there are two modes for N-6 to regulate RXRα interactions with other proteins. One is to regulate the interaction strength, and the other one is to regulate the interaction manner. The regulation mode of RXRα interaction by N-6 should be determined by the interacting domains of both RXRα and its interacting partners such as TR3 as well as other interacting proteins in the RXRα-formed complex such as co-activators and co-repressors in the RXRα/TR3 complex.

TNFα is a double-edged sword in tumor development (Balkwill, 2009; Waters et al., 2013). It stimulates caspase8-dependent apoptosis, and/or promotes survival mainly through PI3K/AKT and NFκB pathways (Mocellin and Nitti, 2008; Balkwill, 2009; Waters et al., 2013). In most cases, TNFα acts as a promoter rather than a killer in tumor cells and tissues because of its abnormally elevated survival functions (Mocellin and Nitti, 2008; Balkwill, 2009; Waters et al., 2013). To take the advantage of TNFα’s apoptotic functions in cancer treatment, its survival signals in cancer cells need to be blocked. Our previous discovery provides a potential target—tRXRα for small molecules targeting to inhibit TNFα survival signals. Our current study presents several lines of evidence that N-6 could target tRXRα to convert TNFα signal from survival to death. First, N-6 strongly inhibited TNFα-induced AKT activation in cancer cells (Fig. 5A and 5B). Second, N-6 and TNFα synergistically induced caspase-8 activation and cancer cell apoptosis (Fig. 6B–E). Third, the functions of N-6 on AKT inhibition and apoptosis-induction with TNFα were dependent on RXRα/tRXRα expression (Figs. 5B and 6E). Forth, N-6 inhibited the interactions of tRXRα and p85α (Fig. 5C and 5D).

Taken together, our results identify a new RXRα antagonist with abilities to modulate TR3 functions indirectly by binding to RXRα and to convert TNFα signal to induce cancer cell apoptosis by targeting tRXRα.

## Materials and methods

### Reagents

NSC-640358 was provided by the NCI/DTP Open Chemical Repository and the DTP website address is http://dtp.cancer.gov.Fermentas TurboFect *in vitro* transfection reagent, DharmaFECT 1 transfection reagent, Gaussia-Dura Luciferase Glow Assay Kit, goat anti-rabbit and anti-mouse secondary antibody conjugated to horseradish peroxidase from Thermo Fisher Scientific, Inc. (Waltham, MA, USA); CD3254, UVI3003, anti-AKT1 (C-20, sc-1618), anti-c-Myc (9E10, sc-40), anti-RXR (ΔN197, sc-774), anti-RXR (D20, sc-553) from Santa Cruz Biotechnology (Santa Cruz, CA, USA); anti-caspase-8 (1C12, #9746), anti-phospho-AKT (Ser473) (D9E, 4060) from Cell Signaling Technology (Boston, MA, USA); anti-poly (ADP-ribose) polymerase (PARP, 556494) from BD Biosciences (San Diego, CA, USA); 9-*cis*-Retinoic Acid, anti-β-actin antibody from Sigma (St. Louis, MO, USA); anti-PI3 kinase (p85α, 04-403), polyvinylidene difluoride membranes from Millipore (Billerica, MA, USA); TR-FRET Retinoic X Receptor alpha coactivator assay kit (PV4797) from invitrogen (Carlsbad, CA, USA); rhTNF-α (Tumor Necrosis Factor-α, Human, Recombinant) from Promega (Beijing, China); enhanced chemiluminescence reagents from GE Healthcare (Buckinghamshire, UK) and a cocktail of proteinase inhibitors from Roche (Meylan, France); [^3^H]9-*cis*-Retinoic Acid from PerkinElmer (Boston, MA, USA) were used in this study.

### Cell culture and transient transfection

MCF-7 breast cancer cells, HCT116 colon cancer cells, HeLa cervix cancer cells, H292 lung cancer cells, A549 lung cancer cells, HEK293T cells, and CV-1 cells were obtained from American Type Culture Collection (ATCC) and cultured in Dulbecco’s modified Eagle’s medium containing 10% fetal bovine serum (FBS). All cell lines used were passaged in our lab for fewer than 4 months after resuscitation, and were used at the fifth through tenth passage in culture for this study. Transient transfection was performed using TurboFect according to the instructions of the manufacturer in regular growth medium.

### Fluorescence ligand binding assay

A dilution series of the compound N-6 in potassium phosphate buffer (10 mmol/L, pH 7.50) was made on a 96-well polypropylene plate, which was subsequently transferred onto a 96-well, half-area UV-transparent quartz plate in duplicate, 50 μL/well. The stock solution of the protein RXRα ligand binding domain was diluted into potassium phosphate buffer to reach 0.60 μmol/L, which was immediately transferred onto the quartz plate, 10 μL/well. The fluorescent intensities of the compound were acquired on a FlexStation 3 Microplate Reader (Molecular Devices, Sunnyvale, CA). In order to minimize the interference from the intrinsic fluorescence of Trp residues, time-resolved fluorescence mode was used. Data analyses were performed with GraphPad Prism 5.0d.

### Mammalian one-hybrid assays

MCF-7 cells seeded in 48-well plates were transiently cotransfected with the luciferase reporter plasmid pG5-Gaussia (40 ng), pBind-receptors (40 ng), and β-Gal plasmid (2 ng). Twenty-four hours after transfection, the medium was replaced by medium containing a respective ligand and/or a testing compound. After 18 h, the medium was collected and assayed by using Gaussia-Dura Luciferase Glow Assay Kit (Pierce). Cells were washed, lysed and assayed for β-Gal activity. Transfection efficiency was normalized to β-Gal activity.

### Mammalian two-hybrid assays

MCF-7 cells seeded in 48-well plates were transiently cotransfected with the luciferase reporter plasmid pG5-Gaussia (40 ng), pBind-TR3-LBD (40 ng), pACT-RXRα and β-Gal plasmid (2 ng). Twenty-four hours after transfection, the medium was replaced by medium containing a respective ligand and/or a testing compound. After 18 h, the medium was collected and assayed by using Gaussia-Dura Luciferase Glow Assay Kit (Pierce). Cells were washed, lysed, and assayed for β-Gal activity. Transfection efficiency was normalized to β-Gal activity.

### CAT assays for NR response elements

CV-1 cells seeded in 24-well plates were transiently cotransfected with one of the luciferase reporter plasmids pBLCAT2-TREpal (100 ng)/pBLCAT2-βRARE (100 ng)/pBLCAT2-NurRE (100 ng), one or two NR expressing plasmids pCMV-Myc-RXRα (20 ng)/pCMV-Myc-RARγ (20 ng)/pCMV-Myc-TR3 (20 ng), and pCMV-Myc-β-Gal (5 ng) as internal reference. Twenty-four hours after transfection, the medium was replaced by medium containing a respective ligand and/or a testing compound. After 18 h, cells were harvested and assayed for CAT and β-Gal activity. Transfection efficiency was normalized to β-Gal activity.

### TR-FRET RXRα coactivator assays

Invitrogen’s LanthsScreen time-resolved fluorescence resonance energy transfer (TR-FRET) assay was used. Retinoic X Receptor alpha coactivator assay was conducted according to manufacture’s protocol. The TR-FRET ratio was calculated by dividing the emission signal at 520 nm by the emission signal at 495 nm.

### SPR measurements

The binding kinetics between RXRα-LBD and N-6 was analyzed at 25°C on a BIAcore T200 machine with CM5 chips (GE Healthcare). PBSP was used for all measurements. A blank channel was used as negative control. About 10,000 response units of RXRα-LBD were immobilized on the chip. When the data collection was finished in each cycle, the sensor surface was regenerated with Glycine-HCl 2.5. A series of concentrations of N-6 ranging from 3.125 to 100 μmol/L were applied for experiment. Sensograms were fit globally with BIAcore T200 analysis using 1:1 Langumuir binding mode.

### siRNA and transfections

siRNAs against RXRα (SASI-HS01-00097639) and control siRNA (SIC001) used were from Sigma. Cells were transfected with 5 μL aliquot of 20 μmol/L siRNA/well in six-well plates using DharmaFECT 1 transfection reagent, according to the manufacturer’s recommendations.

### Western blotting

Cell lysates were prepared by lysing cells with lysis buffer containing 25 mmol/L Tris, 150 mmol/L NaCl, 1 mmol/L EDTA, 1% NP-40, 5% glycerol, pH 7.4 with a cocktail of proteinase inhibitors on ice for 30 min. Equal amounts of the lysates were electrophoresed on an SDS-PAGE gel (8% or 12%) and transferred onto polyvinylidene difluoride membranes, which were then blocked with 5% nonfat milk in TBST [50 mmol/L Tris-HCl (pH 7.4), 150 mmol/L NaCl, and 0.1% Tween 20] for 1 h, incubated with various primary antibodies overnight at 4°C and incubated with secondary antibodies for 1 h at room temperature. Immunoreactive products were detected by chemiluminescence with an enhanced chemiluminescence system.

### Co-immunoprecipitation (Co-IP) assays

For Co-IP assay, cells grown in 10-cm dishes were transfected with various plasmids for 24 h. After transfection and treatments, cells were harvested. Cell lysates were incubated with the appropriate antibody for 2 h, and subsequently incubated with protein A-Sepharose beads for 2 h. The protein-antibody complexes recovered on beads were subjected to Western blotting using appropriate antibodies after separation by SDS-polyacrylamide gel electrophoresis.

### Ligand-binding competition assays

The His-tagged human RXRα-LBD (223–462) was incubated in tubes with unlabeled 9-*cis*-RA or different concentrations of compounds in 200 μL binding buffer [0.15 mol/L KCl, 10 mmol/L Tris·HCl (pH 7.4), 8% glycerol, 0.5% CHAPS] at 4°C for 1 h. [^3^H]-9-*cis*-RA was added to the tubes to a final concentration of 7.5 nmol/L and final volume of 300 μL then incubated overnight at 4°C. The RXRα-LBD was then captured by nickel-coated beads. Bound [^3^H]-9-*cis*-RA was quantitated by liquid scintillation counting.

### Colony formation assay

HeLa cells were seeded in 6-well plate (500 cells/well) for 3 days, and were treated with N-6 (5 μmol/L) and TNFα (10 ng/mL) alone or in combination in 1% serum medium for 3 days. Cells were fixed with 4% paraformaldehyde. Colonies were stained with 0.1% crystal violet and counted.

### Tunel staining

MCF-7 cells were fixed in 4% paraformaldehyde and permeabilized with 1% Triton X-100 in phosphate-buffered saline. Cells were blocked using 3% H_2_O_2_ (80 mL methanol + 10 mL ddH_2_O + 10 mL 30% H_2_O_2_) for 10 min. TUNEL reaction mixture was obtained by adding terminal deoxynucleotidyl transferase and Biotin-11-dUTP. Each sample was then incubated with TUNEL reaction mixture in a humidified chamber at 37°C in dark for 60 min, and then labeled with Streptavidin-HRP. DAB staining was used for coloration. Nucleus was stained by hematoxylin.

### Virtual ligand screening

Virtual ligand screening (VLS) of approximate 200,000 compound library of the Developmental Therapeutics Program (DTP) NCI/NIH (http://dtp.nci.nih.gov) was performed using Q-MOL molecular modeling package (Q-MOL L.L.C., San Diego, CA, USA; www.q-mol.com) (Shiryaev et al., 2011; Remacle et al., 2012; Shiryaev et al., 2012). The Optimized Potential for Liquid Simulations (OPLS) all atom force field (Jorgensen et al., 1996) is uniformly utilized within the Q-MOL program. The ligand docking simulations were conducted using the RXRα crystal structure coordinates from 3FUG PDB. The docking site was prepared as an area encompassing both the ligand binding pocket and the peptide cofactor binding site. The protein molecule preparation included adding of hydrogen atoms and the assignment of the standard OPLS atom types. To increase the speed of calculations and to incorporate implicitly the flexibility of RXR, the protein molecule was treated as a set of grid-based potentials accounting for the relevant protein-ligand interactions. The ligands were docked into the grid-based potentials using the Monte Carlo simulation in the internal coordinate space as implemented in the Q-MOL program. The preparation of each ligand for docking simulation initially included an automatic OPLS atom type assignment and conversion of the 2D sketch-like models (input as the MDL MOL format) into the 3D molecular models. The full-atom ligand structure was then minimized using the Q-MOL small molecule minimization protocol. The protocol combines minimization in both internal and Cartesian coordinates to properly optimize the rotable bonds of a small molecule. In the course of VLS, the compounds were minimally filtered by applying lower molecular mass cut-off of 220 Da, a polyphenols sub-structure filter, and a filter detecting chlorine atoms attached to aliphatic carbons. Because of the stochastic nature of the Q-MOL docking protocol, each ligand was docked at least three times. The best energy conformers with the lowest binding energy were then selected. To differentiate between the true and false binders, Q-MOL VLS uses a proprietary protein-ligand binding energy evaluation function. This function is based on the re-parameterized OPLS force field, and, in addition to the protein-ligand interactions, it accounts for the internal energy change of the docked ligand. Upon completion of VLS, the hits were ranked by relative binding energy, and the best (lowest) energy hits were then selected. The selected hits were visually inspected and the highly symmetric or heavily halogenated ligands and the high molecular weight compounds were discarded and not analyzed further. Out of 100 predicted hits, only 11 were selected and ordered from NCI DTP for *in vitro* testing.

### Modeling of protein-ligand complex

The structure of RXRα was retrieved from crystal complex LG100754-RXRα (PDB entry: 3a9e). Glide was used to study the interaction of ligand-RXRα complex (LG100754 and N-6) in the suite of Schrodinger with default docking parameter settings. The interaction image was produced by the Pymol software.

### Statistical analysis

Data were expressed as mean ± SD. Each assay was repeated in triplicate in three independent experiments. Statistical significance of differences between groups was analyzed by using Student’s *t* test. *P* < 0.05 was considered significant.
